# A Critical Appraisal of Probiotics for Mastitis Control

**DOI:** 10.3389/fvets.2018.00251

**Published:** 2018-10-10

**Authors:** Pascal Rainard, Gilles Foucras

**Affiliations:** ^1^ISP, INRA, Université de Tours, UMR 1282, Nouzilly, France; ^2^IHAP, Université de Toulouse, ENVT, INRA, UMR1225, Toulouse, France

**Keywords:** probiotics, mastitis, dairy ruminants, lactic acid bacteria, mammary epithelium, immune response

## Abstract

The urge to reduce antimicrobials use in dairy farming has prompted a search for alternative solutions. As infections of the mammary gland is a major reason for antibiotic administration to dairy ruminants, mammary probiotics have recently been presented as a possible alternative for the treatment of mastitis. To assess the validity of this proposal, we performed a general appraisal of the knowledge related to probiotics for mammary health by examining their potential modes of action and assessing the compatibility of these mechanisms with the immunobiology of mammary gland infections. Then we analyzed the literature published on the subject, taking into account the preliminary *in vitro* experiments and the *in vivo* trials. Preliminary experiments aimed essentially at exploring *in vitro* the capacity of putative probiotics, mainly lactic acid bacteria (LABs), to interfere with mastitis-associated bacteria or to interact with mammary epithelial cells. A few studies used LABs selected on the basis of bacteriocin production or the capacity to adhere to epithelial cells to perform *in vivo* experiments. Intramammary infusion of LABs showed that LABs are pro-inflammatory for the mammary gland, inducing an intense influx of neutrophils into milk during lactation and at drying-off. Yet, their capacity to cure mastitis remains to be established. A few preliminary studies tackle the possibility of using probiotics to interfere with the teat apex microbiota or to prevent the colonization of the teat canal by pathogenic bacteria. From the analysis of the published literature, it appears that currently there is no sound scientific foundation for the use of probiotics to prevent or treat mastitis. We conclude that the prospects for oral probiotics are not promising for ruminants, those for intramammary probiotics should be considered with caution, but that teat apex probiotics deserve further research.

## Introduction: definition of probiotics and potential relevance to mastitis control

Bovine mastitis is a major economic and welfare problem in dairy farms because of the high incidence of clinical mastitis and prevalence of subclinical mastitis (1–3). Mastitis represents the main reason for antibiotic use for cows, although the proportion of critically important antibiotics remains low compared to other important diseases in cattle (4). The urge to reduce the use of antimicrobials has prompted a wave of studies to find alternatives, and dairy farming is an active field of research in this respect. Alternatives to conventional antimicrobials have been sought for mastitis treatment, such as herbal products, homeopathy, probiotics, and for mastitis prevention notably through genetic selection or vaccination (5–8). Among possible interventions, the use of probiotics is attracting much attention. According to the Food and Agriculture Organization of the United Nations and the World Health Organization, probiotics are “live microorganisms that, when administered in adequate amounts, confer a health benefit on the host” (9, 10). This is a broad definition, inclusive of a variety of microbes and usages. A few criteria that more precisely define probiotics have been proposed: the microbes have to be viable, defined at the species level, safe for intended use, and they need to demonstrate health benefits when used in adequate amounts (9, 11).

A wealth of studies suggests that probiotics have great potential as a tool for improving health and wellbeing. They have established the interest in the probiotic concept and the use of probiotic products in specific settings, such as atopic dermatitis in infants, necrotizing enterocolitis in premature infants, or less specific settings such as the reduction of gut discomfort or antibiotic-associated diarrhea (12). On the other hand, the misuse of the term *probiotic* has become a major issue, and insufficiently rigorous studies have given rise to misleading claims (9).

Trials of probiotics in breast-feeding women or dairy cows have sparked claims of effectiveness for preventing or treating mastitis (13–16), and commercial preparations have been advertised and marketed. Some of these commercial claims have been criticized as ill-grounded (17), for reasons exposed thereafter. Clearly, this is a topic of very active research but also of misuse, calling for clearer guidelines for defining probiotics.

Different categories of probiotics have been proposed by a consensus group (9): probiotics can be with or without any specific health claims. Two common benefits are often associated with the use of probiotics: a healthy digestive tract and a healthy immune system. “Supporting a healthy immune system” is a broad benefit that needs to be specified and demonstrated at the strain level, as is the extension of the notion of use of probiotics from the digestive tract to the reproductive tract, oral cavity, lungs, skin and the gut-brain axis (9). If a probiotic is associated with a specific indication for treatment or prevention of disease, it falls in the category of probiotic drugs (9). Accordingly, the probiotics that claim to prevent or treat mastitis are probiotic drugs, and thus need appropriate trials to meet the regulatory standards for drugs.

An important issue is to define what an appropriate level of evidence for determining a health benefit for probiotics is. According to the consensus group, some of the requirements for a causality relationship between the use of a probiotic and the claimed health benefit are: a temporal relationship; dose response; replication of findings; specificity of association; cessation of exposure; consideration of alternative explanations; biological plausibility; and consistency with other knowledge (9).

Up to now, the primary target of probiotics has been the digestive tract and the gut microbiota. Other sites such as the reproductive tract or the skin have also been considered. The aim of this review is to examine whether the probiotic concept fits the mammary gland (MG) biology, and whether mastitis prevention or treatment is amenable to probiotic intervention. To delineate the possible use of probiotics in the context of mastitis and benefit for the MG, we examined the possible effects of probiotics on MG infections by considering the likely mechanisms of action, taking into account the current knowledge of host/bacteria interactions within the MG. Then we appraised the published literature on the use of putative probiotics in the mastitis setting, although we did not try to perform a meta-analysis because of the small number of reports and their experimental and reporting diversity.

## Mechanisms of action and how they could apply to the MG

There are several categories of possible mechanisms underlying the effects of probiotics (11, 18):

1. The modulation of the composition and activity of the indigenous microbiota, which can result from direct antagonism through production of metabolites or bacteriocins, or competitive exclusion through competition for nutrients.
2. The enhancement of epithelial barrier function, involving either the improvement of the tight junction efficiency or the renewal of epithelial cells, and the induction of antimicrobial peptides (AMPs).
3. The modulation of the immune system in general. Through interactions with monocytes, macrophages, and dendritic cells and training of innate immunity, probiotics may also modulate the balance of helper T cells and thus impact on adaptive immune responses. The interactions with the immune system are very diverse and frequently strain-specific (19).
4. The modulation of systemic responses, for example through endocrine modulations or central nervous system via signaling mediators.

We will examine how these mechanisms could apply to the control of mastitis, i.e., the beneficial modulation of the response of the MG to infection by pathogenic bacteria, except the modulation of systemic responses, for which no data are available. Lactic acid bacteria (LAB) are the bacteria most often used as probiotics, because of their long tradition of safe use in the food industry and the presence of some genera in the “healthy microbiota.” These Gram-positive Firmicutes are considered as GRAS (Generally Recognized as Safe) organisms, and most of them belong to the genera *Lactobacillus, Lactococcus, Bifidobacterium*, and *Streptococcus*. LABs are auxotrophic bacteria adapted to nutrient-rich environments such as milk, in which they can grow fast and reach high concentrations. They owe their name to the fermentation of carbohydrates to lactic acid. Their harmlessness has been established on the basis of their being well-tolerated when administered via the oral route. It is worthy of note that the notion of a safe microorganism is relative to the host and organ, and not only to the microorganism. A microorganism that is safe when administered per os may have detrimental effects when administered via another route. As far as the MG is concerned, several of the LAB genera are classified as MG pathogens, even “major pathogens,” such as several *Streptococcus* and *Lactobacillus* species (20, 21).

## Probiotic effects through microbe-microbe interactions

### Indirect interactions of probiotics with the local microbiota

The most common formulations of probiotics are fresh fermentation products or lyophilized bacteria administered via the oral route. The most used and studied bacteria are LAB of the *Lactobacillus* and *Bifidobacterium* genera, which are present in the healthy human gut (22, 23). Many of the beneficial effects of probiotics are dependent on the indigenous microbiome. Oral probiotics are live and metabolically active bacteria that exert their beneficial effects partly by interacting with the gut microbiota, through restoration of microbial homeostasis or correction of dysbiosis (23). To operate in the MG, this mode of action supposes the existence of a mammary microbiota.

Up to recently, the MG has been considered a sterile organ, but this view is being challenged by studies that found bacterial DNA in milk. This finding was interpreted as evidence that commensal microbial communities are established in the MG of breast-feeding women or dairy cows (24, 25). Particular strains of *Lactobacillus* isolated from human milk have been used as oral probiotics to treat staphylococcal mastitis (26). Bacteria from the two strains used were found in the milk of 6 out of 10 treated women, and mastitis symptoms were not visible at 30 days post-treatment, whereas they were observed in the control group. The authors concluded that the treatment was efficient, but this conclusion was questioned on the basis of several methodological issues related to the definition of mastitis and cure criteria (17). Of particular note, the shedding of *S. aureus* was reduced from 10^4^-10^5^ to 10^2^-10^3^ cfu/mL in the milk of the treated group, a criterion of cure that is not sufficient for a staphylococcal infection or for bovine mastitis.

On the basis of several studies relying on the 16S rRNA gene characterization, bovine mastitis is proposed to result from dysbiosis, i.e., an imbalance between the “healthy” microbiota of the gland and mastitis-causing bacteria (25). Accordingly, correction of this dysbiosis with probiotics is proposed as an alternative to the use of antimicrobials. Up to now, there has been no published evidence that the administration of probiotics to cows have modified the mammary microbiota. In fact, there are a number of observations that cast doubt on the mere existence of an intramammary microbiota [reviewed in (27)]: the udder epithelium is not protected by a mucus that concentrates secretory IgA antibodies or AMPs; there is no sub-epithelial lymphoid formations revealing constant interactions with a microbiota; milk is a rich culture medium for LAB, with a limited capacity to impair the growth of many bacterial species; the MG epithelium is poorly tolerant to Microbe-Associated Molecular Patterns (MAMPs); and the systematic intramammary administration of antibiotics at drying-off, which should be detrimental for the mammary microbiota, is very effective to control MG infections. Above all, the existence of a community of living bacteria in healthy MGs has not been documented.

If we exclude the correction of mammary microbiota dysbiosis, other modes of action can be considered, such as direct probiotic-pathogen interactions.

### Direct interaction of probiotics or colonizing bacteria with the pathogen

Several probiotic bacteria produce a variety of antimicrobial compounds, such as lactic acid, short-chain fatty acids, hydrogen peroxide, nitric oxide, and bacteriocins, all of which could potentially inhibit pathogenic bacteria (18). Bacteriocin production in particular has attracted much interest, and can be considered an important probiotic trait (28). Bacteriocins are peptides produced by bacteria. They are active against other bacteria, against which the producers have a specific immunity system, and they have distinct mechanisms of action that differ from those of antibiotics (29). Bacteriocins have an ecological function allowing the producers to compete with bacterial competitors in an ecosystem, and as such they can contribute to probiotic functionality in several ways: as colonizing facilitators, as AMPs, and as signaling agents for cross-talk with microbes or host cells (28). When acting as antimicrobial agents, bacteriocins produced by Gram-positive bacteria usually have little activity if any against Gram-negative pathogens. To deal with the latter bacteria, Gram-negative bacteriocin producers, such as *E. coli*, have greater potential than LAB (30). Another important consideration is that an antimicrobial compound that showed activity *in vitro* may not necessarily do so *in vivo*, because active production *in vitro* does not ensure a production *in vivo* sufficient to elicit a significant effect in the targeted environment. Consequently, direct administration of bacteriocins may be more effective than probiotics (31).

In relation to the MG, bacteriocin- or hydrogen peroxide-producing LAB have been isolated from the teat canal and milk of dairy cows (32, 33). Most producers belong to the *Streptococcus, Enterococcus*, or *Pediococcus* genera. These bacteria may be harmful for the MG, which would preclude their use as intramammary probiotics. Yet, as the authors suggest that some strains are members of the teat canal microbiota, their use as teat canal probiotics could be envisaged. Other bacteria colonizing the teat canal of cows have been associated with competitive exclusion of pathogens: it has been reported that *Corynebacterium bovis* could reduce the incidence of new infections by mammary pathogens (34–36). This level of protection could be related in part to the recruitment of neutrophils into the milk of infected quarters (37). It is worth noting that *C. bovis*, although considered a minor pathogen for the MG, can colonize the MG lumen (35). These bacteria are occasionally associated with a high proportion of clinical (usually mild) mastitis and is a reason for culling (38), and usually causes increases in milk somatic cell count (SCC) (39), at levels that are not acceptable in the modern era of dairying. Accordingly, it can be anticipated that teat canal probiotics would occasionally be able to reach the lumen of the mammary gland, with the same consequences as those due to intramammary probiotics.

## Enhancement of the epithelial barrier function

The interplay of probiotics with the epithelium has been particularly studied in the intestine, and it has been shown that probiotics are important mediators of intestinal barrier function and integrity (19, 40). Probiotics may exert beneficial effects on the intestinal epithelium in different ways:

1. By improving the mucus layer thickness through stimulation of the mucin-secreting goblet cells
2. By stimulating the production of antimicrobial peptides (defensins and cathelicidins) by epithelial cells and particularly Paneth cells
3. By enhancing tight junction integrity

The mucus layer can limit bacterial movement and contact with epithelial cells, maintain efficient concentrations of secretory IgA and antimicrobial peptides on the surface of the epithelium, and may serve as a substrate for some microbiota members, either for adherence or for nutrition (41).

In the MG, these modes of action are limited by the absence of a mucus layer lining the epithelium. The MG epithelium does not host specialized cells homologous to goblet or Paneth cells. As a consequence, bacteria are potentially in direct contact with the epithelium, and can interact with the cells that make up the epithelium lining, i.e., mammary epithelial cells (MEC) and the associated intraepithelial leucocytes. All probiotics can interact with the pattern recognition receptors (PRRs) of the innate immune system. Probiotic bacteria, commensals and pathogens share most of their MAMPs. The attributes that distinguish these different classes of bacteria are subtle variations in the composition and structure of their MAMPs, the presentation of these compounds at the bacterial surface, and the presence of virulence factors (19). The interaction between probiotics and epithelia depends both on the combination of MAMPs and the expression of PRRs by epithelial cells and the intraepithelial leukocytes such as dendritic cells (DCs) or other antigen-presenting cells. The intestinal epithelial cells have a limited expression of the Toll-like receptors (TLRs) TLR2, TLR4, or TLR5, and they tend to express high levels of TOLLIP, an inhibitor of TLR signaling (42). These limitations are useful to prevent the intestine from overreacting to the large amounts of MAMPs that are generated in its lumen. Other elements, such as the presence of a thick mucus with high concentrations of sIgA, contribute to reducing the stimulation of the epithelium. The skin is prevented from having a strong reaction to its microbiota by the keratinized stratus of its epithelium (43). By contrast, the MG is not a mucosal organ, and its lumen is lined by simple columnar bi-layered epithelium (44).

The innate immune signaling in the MG has been the subject of many studies. Many experiments, both *in vitro* with cultured MECs, and *in vivo* by intramammary instillation of purified or synthetic MAMPs, killed or viable bacteria, have established the reactivity of the MG to a variety of pathogens and putative probiotics. MECs express TLR1, TLR2, TLR3, TLR6, TLR9, but little if any TLR5 (45–47). They seem to express these TLR at a moderate basal level, which can augment in response to PRR stimulation or infection. Another limitation to reactivity is their very little if any CD14 expression at the cytoplasm membrane, even though they may be a source of a significant concentration of soluble CD14 in the milk of healthy glands (48). In the presence of soluble CD14, MECs are very sensitive to the LPS of *Enterobacteriaceae*, but less to lipoteichoic acid (LTA), the commonly used ligands of TLR4 and TLR2, respectively, or to cell-wall peptidoglycan fragments such as diaminopimelic acid and muramyl dipeptide, although they express the cognate receptors NOD1 and NOD2 (47, 49). When stimulated, MECs produce a variety of chemokines that recruit blood leucocytes to mammary tissue and milk and thus contribute to the initiation of an inflammation characterized by a strong influx of neutrophils, i.e., a neutrophilic inflammation (46, 50–53).

LABs possess the MAMPs to which the MG reacts. The inflammatory response of the MG depends on the type of intruding bacteria: coliform bacteria elicit stronger and quicker responses than staphylococci and streptococci (54, 55). The MAMPs and the surface structure of the bacteria, and specifically the accessibility of the molecule or molecular domain to the corresponding PRR, are of prime importance. The lipid moiety of LTA and LPS interacts with the PRRs, implying that these two MAMPs need to be shed from the bacterial surface for TLR engagement. For instance, it has been shown that *Streptococcus uberis* LTA is an agonist of MEC PRRs, whereas whole streptococci induce hardly any response (56). This may well apply to LAB species, which may not strongly stimulate MECs *in vitro* (57). It is noteworthy that the response of the MG to the instillation of MAMPs can be stronger than expected on the basis of *in vitro* experiments with MECs (49). This probably results from the cooperation of intraepithelial cells (DCs and macrophages) with MECs to sense and react to bacterial MAMPs, as the macrophage reaction to mastitis-associated bacteria is stronger than that of MECs (58). Thus, the absence of an MEC response to LAB, as established *in vitro* in a screening test for harmless bacteria, may not predict an absence of a response *in vivo*. In fact, *S. uberis, Enterococcus* spp., and *Lactococcus lactis* are MG pathogens that are able to induce clinical mastitis when inoculated (20, 54, 59).

MECs can produce AMPs and other antimicrobial compounds when stimulated *in vitro* by MAMPs or *E. coli* (60–64). Cathelicidins are found in the milk of inflamed MGs, probably originating from both MECs and recruited neutrophils (65–67). The dilution and scavenging effects of milk components are likely to limit their efficiency. Intense production would be needed, which would require an intense inflammatory and immune response. In fact, heat-stable bactericidal activity, probably due to AMPs produced by MECs and released by neutrophils, can be found in mammary secretions in the case of peracute mastitis (68). There is little information concerning the induction of AMPs by LAB, but it is unlikely that a probiotic that would be safe for the MG could act on pathogens by the mediation of host AMPs. An anatomic site where AMPs could operate is the teat canal mesh-like matrix of keratin that lines the teat canal and seals the canal between milkings and during the dry period (69, 70).

There is no information on the effect of probiotics on MEC tight junctions, the protein complex that forms a relatively impermeable barrier between adjacent cells. There is also no information on the capacity of probiotics to influence the apoptosis and renewal turnover of MECs.

Overall, it appears that LABs and other potential probiotics are very likely to elicit clinical forms of mastitis when introduced within the MG. There can be exceptions, because marked species and strain-dependent variations in pro-inflammatory capacity exists among LAB. As a rule, the reactivity of the MG to MAMPs would preclude the use of LABs as preventive probiotics, but leaves open the possibility of therapeutic use, at the price of a clinical episode.

## Modulation of the immune response

Probiotics may elicit immunomodulatory effects through interactions with the epithelium, especially in organs that are not densely populated by a commensal microbiota, whereas indirect interactions through modulation of massive endogenous microbiota (as in the colon) may be more important (71). Several probiotic effector molecules have been identified. LTA is an important pro-inflammatory molecule of Gram-positive bacteria, and as such is suggested to be an undesirable attribute for bacteria used as probiotics (72). However, mutants of *Lactobacillus* strains that produce LTA variants have induced fewer pro-inflammatory molecules and more anti-inflammatory cytokines *in vitro* (73). Other MAMPs such as proteins produced by certain strains of *L. plantarum* or *L. salivarius* can modulate the inflammatory response (18). Immune modulation can be induced by probiotics through the dampening of inflammatory pathways (NF-kB or histamine-dependent pathways), or the reprogramming of epithelial-associated T cells such as helper T cells that produce the IL-17 cytokine acting on epithelial cells (Th17 cells), or the regulatory Treg cells that counteracts pro-inflammatory signals (18, 74–76). Probiotics are also supposed to modulate the balance of helper T cells by modifying the regulation of the pathways linked to the different helper T cells (Th1/Th2/Th17) and the regulatory Treg cells (73).

Our limited knowledge of the homeostatic and protective helper T lymphocyte balance in the MG makes it difficult to predict the consequences of the modulation of this arm of the immune defense by mammary probiotics. This modulation can be useful in an organ subjected to constant stimulation by a sizable microbiota as in the intestine, which is a major site of Th17 cell generation and population (76). At this site, the epithelium may favor a certain degree of immune tolerance (75). The mammary gland is not constantly subjected to inflammatory stimuli. It is not obvious that reducing its capacity to react through the dampening of signaling pathways would be beneficial, as this could weaken the immune response to an infection. There are actually published instances when reduction of the reactivity of the MG is detrimental, as shown in the setting of coliform mastitis or more generally with the immune-depression that accompanies the peripartum period (77). The balance of Th17/Treg cells favoring the production of IL-10 over that of IL-17 may aggravate *E. coli* mastitis (78). To tip the balance in favor of Treg cells in the MG may not be advisable. Besides the production of IL-17A, the elicitation of the local production of IFN-γ has been shown to be associated with a reduction of the severity of *E. coli* mastitis (79). Further studies will be necessary to improve our understanding of this important issue. In the meantime, indiscriminately transposing to the MG concepts established for the intestine may lead to unwanted deleterious consequences.

The other way round, an increase in the reactivity of the MG to infection, accompanying a moderate level of inflammation, may be envisaged through the use of probiotic bacteria. Although LABs are the most commonly used probiotics, they may not be the most appropriate bacteria to deal with mastitis. The bacteria most successfully adapted to the MG niche are the coagulase-negative staphylococci (CoNS), which comprise several species frequently associated with the MG (38, 80, 81, 82). Some studies have found that MGs infected by CoNS are less prone to superinfection with more pathogenic bacteria (36, 83, 84). Some of the CoNS strains isolated from milk can produce bacteriocins (85, 86) and thus could be putative mammary probiotics. Yet not all studies have shown a protective effect of *C. bovis* or CoNS on infection by more pathogenic bacteria (87–89), so it is difficult to make a definitive statement about the effect of minor pathogen on the acquisition of major pathogen MG infections (90). The point can be made that protection was mostly evidenced by experimental infections, but usually not under field conditions (91). A plausible explanation is that minor pathogens exert their protective effect mainly through an increase of the SCC, comprising neutrophils and mononuclear leukocytes, cells that constitute a leukocyte barrier to infection (92). This recruitment of leukocytes is uneven with frequent SCC fluctuations, and could sometimes be insufficient to constitute an effective barrier. If bacterial intrusion into the MG takes place at a low SCC ebb, infection could occur. Interestingly, the impact of *C. bovis* or CoNS infections of the MG on milk yield was found to be either slightly detrimental or even favorable (39, 82, 93, 94). It is possible that different CoNS species, and strains within species, have different impacts on MG inflammation and milk yield or composition (80). If the lead of MG minor pathogen is to be followed for the development of mastitis probiotics, species and strain specificity will have to be taken into account. The sought properties of an ideal strain would be low pro-inflammatory activity, capacity to induce very mild sub-clinical but chronic MG infections, and production of bacteriocins effective on major pathogens. The requirement for low bulk milk SCC is likely to constitute a major hurdle for the large scale use of the candidate bacteria.

## Appraisal of published probiotic trials in relation to mastitis control

Probiotics may have different uses in relation to mastitis control. Relative to the lactation cycle, probiotics could be applied at drying-off, in the dry period, around parturition, or during lactation. Relative to the application site or route, the bacteria used as probiotics could be administered by the oral route, intramammarily through the teat canal, or at the teat apex by teat dipping. Mastitis probiotics may also be used to prevent, mitigate or cure MG infections. These different potential uses deserve separate evaluations. In general, the authors reporting preliminary experiments (*in vitro* interactions of probiotic strains with mastitis-associated pathogens or MECs) interpret their data as favorable for use of LABs as probiotics for the MG, the oral administration to breast-feeding women is reported as an effective alternative to antibiotic therapy [but there are grounds for doubts (17)], and the intramammary administration to prevent or treat mastitis in ruminants raises reservations (Table 1).

**Table 1:** Study characteristics of 19 included manuscripts evaluating the potential of probiotics for the prevention or treatment of mastitis.

**References**	**Probiotic species or strain**	**Intervention type**	**Host species or cell culture**	**Time of intervention**	**Application site or route**	**Authors' conclusion**
(95)	*Bifidobacterium* spp.	*In vitro* on cell line	HT29 cell line	NA	*In vitro*	Potential to be ascertained with clinical trial
(96)	*Lactobacillus casei*	*In vitro* on cell line	Bovine MEC MAC-T cell line	NA	*In vitro*	Inhibition of cell invasion by *S. aureus*
(57)	*Lactobacillu*s or *Lactococcus* spp.	*In vitro* on cell line	Bovine MEC MAC-T cell line	NA	*In vitro*	Promising candidate strains for prevention or treatment of mastitis
(97)	*Lactococcus lactis* ssp. *lactis*	*In vitro* on cell line	Bovine BME-UV1 cell line	NA	*In vitro*	Some strains have a potential for treatment of mastitis
(98)	*Lactococcus lactis* LMD 7930	*In vitro* on cell line	Bovine BME-UV1d cell line	NA	*In vitro*	Potential as mastitis probiotics but efficacy and safety to be established
(99)	Lactic acid bacteria (several species)	*In vitro* on primary cells	Bovine teat canal epithelial cells	NA	*In vitro*	Potential of strains to be used at drying-of for prevention of mastitis
(26)	*Lactobacillus salivarius* CECT5713 and *Lactobacillus gasseri* CECT5714	Treatment of mastitis	Human	In lactation	Per os	Efficient alternative to antibiotic therapy for the treatment of infectious mastitis
(13)	*Lactobacillus fermentum* CECT5716 or *Lactobacillus salivarius* CECT5713	Treatment of mastitis	Human	In lactation	Per os	Improvement of clinical condition by probiotics. Alternative to antibiotic therapy. Criticized by (17)
(14)	*Lactobacillus salivarius* PS2	Prevention of mastitis	Human	Late pregnancy	Per os	Reduction in incidence of mastitis
(100)	*Lactococcus lactis* LMG 7930	Preliminary safety experiment	Mouse	lactation	Intramammary	*L. lactis* LMG 7930 induces mastitis, nor safe for the mammary gland
(101)	*Lactobacillus acidophilus* and *Lactobacillus casei*	Treatment of mastitis	Cows	Lactation	Intramammary	Lactobacilli induce mastitis with no effect on the infection prevalence
(16)	*Lactococcus lactis DPC3147*	Treatment of mastitis	Cows	Lactation	Intramammary	As effective as antibiotic therapy for clinical mastitis
(102, 103)	*Lactococcus lactis DPC3147*	Safety experiment	Cows	Lactation	Intramammary	Induces an inflammatory immune response
(15, 104)	*Lactobacillus perolens CRL 1724*	Safety experiment	Cows	Lactation	Intramammary	Induced inflammation as a function of dose
(105)	*Lactobacillus perolens CRL 1724*	Safety experiment	Cows	At drying-off	Intramammary	Stimulation of immune defenses
(106)	*Lactococcus lactis ssp. lactis* LMG 7930	Treatment of mastitis	Ewes	Lactation	Intramammary	Inflammation but no cure of staphylococcal infections
(107)	Lactic acid bacteria	Prevention and treatment	Cows	Lactation	Teat apex (teat dipping)	Transient decrease of somatic cell count

*MEC, mammary epithelial cells; NA, not applicable*.

### Oral administration of putative mastitis probiotics

A few evaluations of oral probiotics have been performed in breast-feeding women (13, 14, 26). The authors, who used lactobacilli isolated from milk, reported that the administered LAB reduced the bacterial counts in milk and the pain score of the treated patients. They stated that the probiotics were an efficient alternative to antimicrobials for the treatment of infectious mastitis during lactation. These studies have been criticized and deemed inconclusive (17). Specifically, in the pioneering study by Arroyo et al. (13), the results of only one follow-up time point (21 days post-treatment) was reported, a long time for someone suffering from mastitis; the assignment of treatments was not blinded, the control patients receiving antibiotic treatment at the discretion of their clinicians; most of the prescribed antibiotics are known to be inefficient to treat *S. aureus* infections, which explained the poor outcome of the control treatment; the definition of mastitis and the recruitment of cases were not clearly stated.

The rationale for the use of the oral route is that lactobacilli and other LABs isolated from human milk have an endogenous origin, being translocated from the gut lumen to the MG lumen by dendritic cells or macrophages along the entero-mammary pathway (108, 109). The generalization of this theory to the MG of dairy ruminants is open to doubt. The existence of an entero-mammary pathway able to seed the mammary gland with live bacteria from the gut microbiota would constitute a formidable and permanent threat to the mammary gland, considering that only a few colony-forming units of enterobacteriacae, staphylococci, streptococci, or enterococci are able to induce a clinical mastitis with a probability near to certainty (110–112). It is fortunate that the entero-mammary pathway is not likely to operate in ruminants (77). Alternatively, the extracorporeal route is likely to explain the recovery of the orally administered probiotics in milk samples. In this scenario, the direct administration of probiotics within the MG would be more efficient.

### Intramammary administration of LABs for mastitis prevention or treatment

The selection of LAB species and strains to be used as putative mammary probiotics is largely based on the isolation from milk or the teat canal, and their *in vitro* assessment of antibacterial activity against mastitis pathogens. Isolation from milk samples is construed as evidence that the bacteria are natural members of the mammary microbiota, thus they are supposedly innocuous commensals that are able to colonize the MG, and are more efficient for the protection of the MG than probiotic bacteria used for other applications (95). Criteria used for the pre-selection of probiotic strains isolated from milk samples are the production of bacteriocins or other compounds interfering with the growth or survival of mastitis pathogens, the absence of known virulence factors or wide antibiotic resistance, and sometimes surface properties as indicators of the propensity to colonize an epithelium site (33). Probiotics used to prevent bacterial spoilage of cheese were also considered as mammary probiotic candidate and the capacity of the selected strain to adhere to and invade MECs was assessed (98).

Preliminary steps before testing in the MG of dairy ruminants made use of MECs in culture, or a mouse mastitis model, with a view to assessing the effect of putative probiotics on the local immune response or the innocuousness of the bacteria and their products (97, 96). What could be sought is the absence of cytotoxic activity and a low or preferably diminished pro-inflammatory response assessed through the limited or reduced production of cytokines and chemokines. The bacteria that fulfill these criteria are considered promising for use as mammary probiotics. This first *in vitro* step is of limited value, because it is highly dependent on experimental conditions that are non-physiological and differ widely from the *in vivo* situation. Also, bacteria that induce little if any response on the part of MECs *in vitro* may well be major pathogens for the MG, as exemplified by *S. uberis* (56, 58). Thus the absence of cytotoxicity or pro-inflammatory activity *in vitro* with MECs is not sufficient to establish the safety and innocuousness of a bacterial strain.

It is known that LABs such as *L. lactis* and several streptococci or enterococci are major pathogens for the MG of dairy ruminants (20, 21, 113). It is worth noticing that the characteristics that distinguish *L. lactis* mastitis isolates from dairy starter strains (higher tolerance to body temperature, lysozyme and bile, wider carbohydrate fermentation capacity and better adhesion to MECs) are considered desirable probiotic attributes (113). According to their pathogenic potential, in most studies, LABs elicited an immune response that was potentially harmful and detrimental to the secretion of milk when infused into the MG. A mouse mastitis model was used to conduct in depth investigations of the immune response induced by the intramammary infusion of a food-grade *L. lactis* strain (100). The authors concluded that the strain used could not be considered safe for the MG. Other studies showed that the infusion of *L. lactis* into the bovine MG induced an intense recruitment of neutrophils, increased concentrations of acute phase proteins in milk, and overexpression of genes encoding the chemokine IL-8 and the pro-inflammatory cytokine IL-1 β (102, 103). However, the pro- or anti-inflammatory effects of probiotics can be strain-specific. This specificity may account for the reported mild and transient inflammation induced by the probiotic *Lactobacillus perolens* CRL1724 (15). Notably, the inoculation of lactating MG was carried out by instilling the bacteria into the teat canal at a depth of 17 mm at a volume of 1 mL, which indicates that part of the inoculum reached the teat cistern and diffused into the MG. There was a dose-effect on the reaction of the MG, 10^9^ CFU causing a clinical episode, whereas 10^3^ or 10^6^ provoked only a transient doubling of the SCC. This suggests that this probiotic is not able to cope with the MG defenses and is promptly eliminated from the lumen of the gland. However, the bacteria were found in milk samples for at least 18 days, suggesting that they persisted in the teat canal. In a subsequent study, the authors assessed the reaction induced by the inoculation of 10^6^ cfu of *L. perolens* CRL1724 at the teat cistern level. They found an infiltration of neutrophils that could reach 4.5 × 10^6^ cells/mL 24 h after inoculation with a return to the baseline of 1 × 10^5^ cells/mL after 7 days, showing that these bacteria induced an inflammatory reaction when introduced into the MG (104). The shedding of the putative probiotic in milk was less than 25 CFU/mL, a value that indicates the low capacity of this strain to resist MG defenses and is compatible with the contamination of the samples as the milk passes the teat canal. Importantly, the colonization of the teat canal was supported by the demonstrated adherence of the bacteria to teat canal epithelial cells obtained by teat canal scraping (15). As this probiotic strain was shown to be able to interfere with the growth of major mastitis pathogens, it could be a candidate for prevention of mastitis as a teat canal probiotic (see below). From all the studies reporting the intramammary infusion of LABs, it can be concluded that those bacteria exert a pro-inflammatory effect on the MG.

To minimize the downsides of LAB infusion into lactating MGs, they could be used at drying-off. A preliminary experiment was performed with *L. perolens* CRL1724, which caused a mild inflammatory response when used at drying-off at the dose of 10^6^ CFU, with associated increase in antibodies in MG secretion and some non-specific increase in lymphocyte proliferation response (105). The authors conclude that the probiotic strain could be used at drying-off as an immune response modifier, but this possibility awaits experimental confirmation. The adherence of the candidate bacteria to teat canal epithelial cells, along with the capacity to co-aggregate with mastitis pathogens or inhibit their growth are deemed valuable properties of potentially valuable strains (99).

The inflammatory reaction induced by intramammary infusion of LAB precludes large scale preventive use in lactating dairy animals, but LAB could be used as a treatment of mastitis, at the cost of a clinical episode. Few trials comparing the efficacy of probiotics to that of antibiotics have been reported. A combination of two LABs (*Lactobacillus acidophilus* and *Lactobacillus casei*) was used to treat subclinical mastitis mainly due to CoNS (101). The LABs were much less efficient than the antibiotic preparation in curing the infected quarters. In two small scale trials, chronic subclinical or clinical mastitis were treated either with a food-grade probiotic (*L. lactis* DPC3147) or a conventional antibiotic preparation (16). The cure rates of the two treatments were similar. Two field studies carried out on ewes were used to assess the capacity of the *L. lactis* LMG7930 to cure staphylococcal (CoNS or *S. aureus*) infections (106). The infused glands developed an inflammatory response, which was accompanied by a transient eclipse in CoNS milk shedding followed by relapse, but no effect on *S aureus* infections. Overall, in the current state of the art, treatment of chronic or clinical MG infections with LABs cannot be recommended (5).

A possible side-effect of the long-standing shedding of LABs into milk is that they alter milk quality and interfere with the transformation procedures involved in the making of dairy products. As LABs used as probiotics share a number of properties with dairy starters, this possibility should be taken into account.

### Topical application of bacterial colonizers at the teat apex

The teat canal is the port of entry of most MG infections. Consequently, the microbiota that colonizes the teat skin, and particularly the teat apex close to the orifice of the teat canal and the teat canal itself, may be of significance in relation to the occurrence of new intramammary infections. This is reminiscent of the interference of the teat canal colonization by *C. bovis* with the incidence of new mammary infections. Mammary quarters shedding *C. bovis* were more resistant to experimental intramammary challenge with *S. aureus* but more susceptible to challenge with *Streptococcus agalactiae* than uninfected quarters (35). This result was not confirmed when quarters were challenged by exposure of the teat orifice to the same pathogens, colonization by *C. bovis* being without effect (34). An observational study involving 74 herds did not show a reduction in new infection rate by mastitis pathogens in quarters or cows shedding *C. bovis*, even though the SCC in the infected quarters (150,000–200,000/mL) was significantly higher than in uninfected quarters (87). In these studies, the capacity of the strains of *C. bovis* to interfere with the mastitis pathogens had not been assessed, but the idea here is that bacteria that inhabit the teat apex or the teat canal may interfere with the colonization and reduce the incidence of MG infections by major pathogens such as *S. aureus*.

By swabbing teat apices, it is possible to collect contaminants from the environment, transient flora, and resident flora. Although washing can reduce the environmental contamination, it is difficult to distinguish the resident from the transient flora. It has been found that a proportion of the bacterial strains isolated from the teat apex of mainly the *Corynebacterium* and *Bacillus* genera can inhibit the growth of mastitis pathogens, more efficiently against Gram-positive *S. aureus, S. epidermidis*, or *Corynebacterium pyogenes* (now *Trueperella pyogenes*) than against Gram-negative *E. coli* or *Klebsiella* (114). The authors suggested that colonization of cattle teats with bacterial strains of the teat skin microbiota that are inhibitory *in vitro* for mammary pathogens could be a prospective tool for the prevention of mastitis.

Exploration of the teat apex microbiota revealed a low bacterial load but a diversity of bacterial genera, among which CoNS were the most prevalent (115). Considering that the teat skin is a potential reservoir of mammary pathogens like *S. aureus*, that colonization of the teat apex by *S. aureus* is a threat to the MG (116) and that LAB can interfere with this pathogen (117), competitive exclusion at this anatomical site could be of interest. Isolation from the teat canal can be construed as evidence that the bacteria are natural members of the mammary microbiota, and thus supposedly innocuous commensals able to colonize the teat entry. As some of the isolated bacteria such as bacteria of the *Bacillus* genus possess inhibitory activity *in vitro* against mammary pathogens, they could have the potential to be used as teat probiotics (118). In a study to assess their potential as probiotics, LABs were isolated from teat canal swabs or foremilk samples of lactating cows (57). Several isolates of the *Lactobacillus* and *Lactococcus* genera were characterized and showed inhibitory activity toward major mastitis pathogens (*S. aureus, S. uberis*, and *E. coli*), adherence and internalization by MECs. They did not activate MECs *in vitro*, and two isolates were even able to reduce the response of MECs to *E. coli*, when used at a 100 to 1 ratio relative to *E. coli*. The lactococci and lactobacilli found on the teat apex or in the teat canal could be part of a resident teat microbiota. In this hypothesis, they should possess characteristics enabling them to colonize the teat apex epithelium, which is a skin epithelium, and to compete with mastitis pathogens for the colonization of this niche. The teat canal epithelium is a stratified cutaneous epithelium, which transitions to the intramammary bi-layered epithelium at the Fürstenberg's rosette formation (44). Thus, the relevance of measuring the adherence, internalization and stimulation activities of teat apex LABs is obvious for cutaneous epithelial cells, but not MECs, unless it is considered that these LABs colonize the intramammary lumen. This has been taken into account to evaluate the capacity of a probiotic strain to colonize the teat canal by using teat canal epithelial cells obtained by teat canal scraping (15). It was found that bacteria adhered to the cells but not to the keratin. This could limit the relevance of this observation, as the teat canal lumen is lined essentially by keratin (119, 120). A limitation of the technique is that the scraped cells expose normally masked components of the teat lining to the bacteria under test, thus the *in vitro* test may not capture the *in vivo* situation.

A potential application of the competitive activity of teat LABs is their use as teat-dip probiotics. An experiment was carried out with a mixture of two probiotic LABs (isolated from pickles) applied as a teat-dip (10^10^ CFU/mL) to two quarters of each of 25 cows, the other two quarters being dipped in a commercial disinfectant product (107). The authors reported a slight and similar decrease in the SCC of the quarters treated with either product over time, but curiously the SCC returned to the pre-treatment level as soon as the treatments ceased. Another curiosity was the shedding in the milk samples of 10^3^-10^5^ copies of *S. aureus, E. coli*, and S*. agalactiae* measured by quantitative PCR, among many other bacteria assimilated to the mammary microbiota. Such concentrations of major pathogens are generally associated with clinical mastitis but are rarely found in association. This unexpected observation is likely to result from the sampling technique and bacterial identification from 3 mL of milk, a method prone to heavy contamination by environmental bacteria and devoid of any methodological controls. Although there is not yet experimental confirmation that this approach is feasible, the topical administration of LAB or other members of the teat microbiota to prevent the colonization of the teat apex by mammary pathogens deserves more serious examination. Technical considerations may hamper the development of teat probiotics: probiotics are live organisms, likely to lose viability and degrade over time. Their manufacturing involves a great number of variables, such as the choice of isolate, the production processes, the long-term series of re-inoculations to prepare new batches, the effect of the culture matrix or environment on the probiotic. This calls for quality-control measures in probiotics manufacturing to retain their original properties (121, 122).

## Concluding remarks and prospects

The question we wanted to answer was: does the probiotic concept fit the mammary gland biology? In other words, is mastitis amenable to probiotic intervention? There are several answers because probiotics can be used in different ways to prevent or treat mastitis, and each way has its peculiarities.

If we consider the level of evidence required for determining a health benefit of probiotics for the MG, it is clear that the assertion that LAB constitute an alternative tool for bovine mastitis prevention is not substantiated by available data. There is a lack of convincing data supporting the benefits of probiotics for the MG. In addition, the requirement for the replication of findings, the consideration of alternative explanations, the biological plausibility and the consistency with other knowledge such as the immunobiology of the MG and the pathogenesis of mastitis, is not fulfilled.

Oral probiotics have been used to treat mastitis in breast-feeding women, and are claimed to be safe and efficient. It appears that there is no robust scientific foundation for this assertion, and there is no sound scientific foundation to support the entero-mammary pathway in dairy ruminants. The development of oral probiotics to treat mastitis does not appear promising. The prospects for oral probiotics are rather in the realm of non-specific health-promoting effects.

It is clear that the preventive use of intramammary probiotics can hardly be envisaged in lactating cows because even a very mild inflammatory response would be an issue at the herd level. Most LAB strains are pathogens for the MG, and their GRAS status, valid for the digestive tract, is not a pass for intrammamary use. It can be claimed that certain probiotic strains could be well tolerated by the MG. The problem then would be the efficiency of such strains as probiotics. We have seen that several of the mechanisms that are suggested to explain probiotics' beneficial effects could not operate in the MG. Others, such as the production of bacteriocins or other antimicrobial compounds, suppose a level of production requiring high concentrations of bacteria either in milk or adhering to the mammary epithelium, a condition that the MG is very unlikely to tolerate. Maybe the closest approximate of an intramammary resident probiotic are chronic infections by CoNS. Even with CoNS, the protection is uneven, and relies very likely on the induced recruitment of leucocytes to the MG. There is no published evidence that the production of bacteriocins within the MG can exert an antibacterial effect on MG pathogens.

A preventive use of intramammary probiotics at drying-off would be of less consequence in terms of induced inflammation. At this phase of the lactation cycle, probiotics could have both a preventive and curative indications. Bacteriocins might contribute to bacterial competition in a dry MG, but to date, there have been no evidence-based findings to support the safety and efficacy of probiotics at drying-off.

If we consider intramammary probiotics with a view to curing intramammary infections, probiotics have a drug status and need to abide by the stringent regulations that govern this class of products (9). The few published studies do not fulfill these criteria (as randomized controlled field trials). Moreover, the few published experiments have not reported encouraging results. Another downside of intramammary probiotics is the inflammatory response they trigger, which would be justified only for probiotics that are noticeably more efficient that the current antimicrobial treatments. For these reasons, the prospects for intramammary probiotics do not appear very promising.

The topical use of probiotics at the teat apex may have a biological plausibility and could fit with the concept of teat apex microbiota. Competition between probiotic bacteria and pathogens at the level of the teat apex or teat canal could be of interest. Nevertheless, teat duct colonization frequently leads to or accompanies infection of the MG, even with *C. bovis* that is reputed to preferentially colonize the teat canal. Despite these reservations, modifying the teat microbiota through teat dipping during lactation or at drying-off may be envisaged. Our knowledge of the teat apex microbiota and its adaptation to the teat canal niche is limited, and needs improvement to effectively develop teat apex probiotics. Considering the importance of the teat canal as the gatekeeper of the MG, research on this crucial defense of the MG against mastitis is amply justified.

In conclusion, it can be suggested that the effectiveness of probiotic bacteria in preventing or treating mastitis of dairy ruminants is not soundly established, and we concur with others that currently probiotics cannot be recommended for this indication (5). Nevertheless, some research leads appear to be of interest with a view to reducing the use of antimicrobials for dairy ruminants.

## Author contributions

PR conceived the design of this manuscript. PR and GF wrote the manuscript.

### Conflict of interest statement

The authors declare that the research was conducted in the absence of any commercial or financial relationships that could be construed as a potential conflict of interest.
